# A Dynamic Bayesian Model for Identifying High-Mortality Risk in Hospitalized COVID-19 Patients

**DOI:** 10.3390/idr13010027

**Published:** 2021-03-18

**Authors:** Amir Momeni-Boroujeni, Rachelle Mendoza, Isaac J. Stopard, Ben Lambert, Alejandro Zuretti

**Affiliations:** 1 Department of Pathology, Brigham and Women’s Hospital, Boston, MA 02115, USA; amomeni-boroujeni@bwh.harvard.edu; 2 Downstate Medical Center, Department of Pathology, State University of New York, Brooklyn, NY 11203, USA; Rachelle.Mendoza@downstate.edu; 3 MRC Centre for Global Infectious Disease Analysis, School of Public Health, Faculty of Medicine, Imperial College London, London W2 1PG, UK; isaac.stopard11@imperial.ac.uk (I.J.S.); ben.c.lambert@gmail.com (B.L.)

**Keywords:** SARS-CoV-2, prognostication, triage, time trends, Markov model

## Abstract

As Coronavirus Disease 2019 (COVID-19) hospitalization rates remain high, there is an urgent need to identify prognostic factors to improve patient outcomes. Existing prognostic models mostly consider the impact of biomarkers at presentation on the risk of a single patient outcome at a single follow up time. We collected data for 553 Polymerase Chain Reaction (PCR)-positive COVID-19 patients admitted to hospital whose eventual outcomes were known. The data collected for the patients included demographics, comorbidities and laboratory values taken at admission and throughout the course of hospitalization. We trained multivariate Markov prognostic models to identify high-risk patients at admission along with a dynamic measure of risk incorporating time-dependent changes in patients’ laboratory values. From the set of factors available upon admission, the Markov model determined that age >80 years, history of coronary artery disease and chronic obstructive pulmonary disease increased mortality risk. The lab values upon admission most associated with mortality included neutrophil percentage, red blood cells (RBC), red cell distribution width (RDW), protein levels, platelets count, albumin levels and mean corpuscular hemoglobin concentration (MCHC). Incorporating dynamic changes in lab values throughout hospitalization lead to dramatic gains in the predictive accuracy of the model and indicated a catalogue of variables for determining high-risk patients including eosinophil percentage, white blood cells (WBC), platelets, pCO2, RDW, large unstained cells (LUC) count, alkaline phosphatase and albumin. Our prognostic model highlights the nuance of determining risk for COVID-19 patients and indicates that, rather than a single variable, a range of factors (at different points in hospitalization) are needed for effective risk stratification.

## 1. Introduction

As global coronavirus disease (COVID-19) deaths exceed 2.5 million [1], predictors of severe disease and mortality are necessary to inform clinical decisions and guide patient care. Efficient COVID-19 transmission, a relatively high infection fatality ratio [2] and underprepared health systems [3] have seen many hospitals exceed capacity [4,5]. In the context of insufficient surge capacity, these predictors, alongside ethical considerations to avoid health inequities [6,7], may save lives through early risk stratification and better resource management [8,9]. Consequently, prognostic models are needed to identify the relative importance of different prognostic factors, their impact on mortality risk and to predict the course of infection of hospitalized patients [10]. Since the beginning of the pandemic, a wide variety of prognostic models have been developed [11,12]. Such models have identified novel predictors of mortality, including a range of socioeconomic variables, demographic variables and biomarkers. In particular, patients who are older [13,14] and have existing comorbidities (such as cardiovascular disease, hypertension and diabetes) [15] are at greater risk of in-hospital mortality. Laboratory markers are also indicative of certain pathologies associated with mortality: including (i) abnormal inflammatory markers (elevated C-reactive protein, ferritin, lactate dehydrogenase and procalcitonin, and lymphopenia) [16], (ii) myocardial injury biomarkers (elevated troponin) [17,18], biomarkers of acute respiratory distress syndrome (ARDS) (hypoxaemia and hypercapnia) [19], (iii) coagulopathy markers (elevations in D-dimer, thrombocytopenia, and prolonged prothrombin time) [20,21,22].

By combining multiple variables into a single analysis, prognostic models can be used to investigate the relative importance of different prognostic factors and evaluate their impact on mortality risk [23]. Existing prognostic models, which quantify the in-hospital mortality risk associated with these biomarkers, have mostly considered test values at a single time-point only (typically presentation) and to a single outcome (typically mortality) at a single maximum follow-up time [11,12]. Throughout the course of hospitalization there are, however, outcome-specific dynamic changes in certain biomarkers. Upward trends in D-dimer, neutrophil-lymphocyte count ratio (NLR), neutrophils, interleukin-6 (IL-6), procalcitonin, amyloid-A protein and C-reactive protein (CRP) have been associated with mortality [24,25], whilst increasing levels of lymphocytes, eosinophils and platelets from admission are indicative of survival [25,26], sustained IL-6 and interleukin-10 (IL-10) (cytokines) and interferon gamma inducible protein 10 (IP-10, a chemokine) are also useful to anticipate disease progression [9].

To quantify dynamic changes in biomarkers on in-hospital COVID-19 mortality risk, studies have either incorporated the difference in biomarker values between presentation and outcome within a Cox model [25], fit individual logistic regressions each day post hospitalization, or used a joint model in which the survival model hazard can vary with longitudinal changes in biomarkers, which are estimated using a mixed effects framework [27,28]. At a given time, hospitalized COVID-19 patients have, however, multiple possible outcomes: (1) discharge, (2) remain in hospital or (3) mortality. Incorporating these competing risks into survival models is challenging, and to facilitate model fitting, some analyses exclude or treat as censored those patients that do not meet the required criteria, such as those experiencing the event of interest, causing a high risk of bias [12,29].

Markov models can be used to model individual trajectories through a finite number of states. Assuming transition probabilities from one state to another depend only on the current state and remain constant over time it is possible to calculate the probability individuals will transition from one state to another, which allows multiple competing risks [30]. We aimed to develop a prognostic Markov model for hospitalized COVID-19 patients which incorporates dynamic laboratory value data along with patients’ admission profiles: allowing us to identify key determinants of risk. A recent comparison of 22 prognostic models found none demonstrated considerably more benefit than using the univariable predictor age [11]. We hypothesize that incorporating dynamic changes in laboratory findings will improve the predictive accuracy of prognostic models.

## 2. Materials and Methods

### 2.1. Case Selection and Data Extraction

Approval for the study was obtained from the State University of New York, Downstate Medical Center Institutional Review Board (IRB#1595271-1).

A retrospective query was performed among patients admitted to SUNY Downstate Medical Center with COVID-19-related symptoms and confirmed Polymerase Chain Reaction (PCR)-positive from early February 2020 until the end of March 2020. Stratified randomization was used to select at least 200 patients who were discharged and 200 patients who died due to COVID-19 complications. Patients whose outcome was unknown were excluded. The outcome for patients was recorded as either “discharged” or “COVID-19 related mortality” (expired). Demographic, clinical history and laboratory data were extracted from the hospital’s electronic health records.

### 2.2. Models of Dynamic Risk

The data were processed to convert them into a form amenable to estimation (see Supplementary Materials (SOM)), resulting in a few individuals and tests being dropped from the analysis (mainly due to missing data). This meant that 475 individuals and 28 laboratory tests were included in our models.

**Figure 1:**
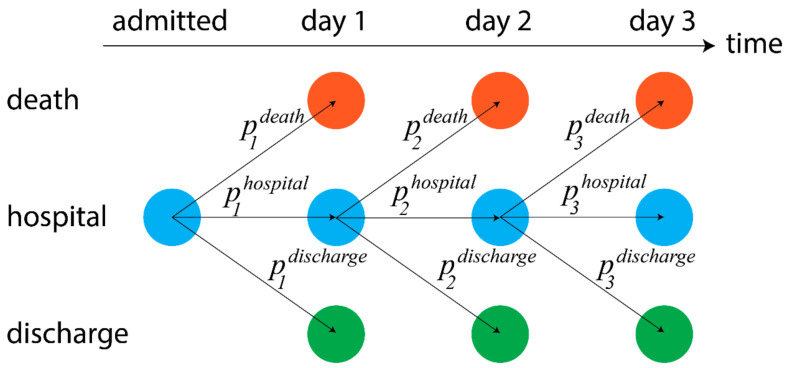
Markov model schematic: colors indicate each of the three distinct states. Arrows indicate potential changes in the patient’s state from one day to the next. These state transitions are influenced by probabilities which, in the model, can vary with patient characteristics, timing of their admission, initial lab values and dynamic changes in values.

Two sets of analyses were conducted: the first was a Markov model, which analyzed the dynamic sequence of observations for each patient throughout their stay and aimed to examine how changes in these variables affected the probability a patient was discharged or died on a given day (Figure 1; SOM). The second was a logistic regression analysis, which aimed to determine those factors most predictive of patient mortality, and not assess dynamic changes in mortality risk. Both models were estimated in a Bayesian framework (details in SOM) and, as such, there is no need for an arbitrary cutoff representing whether a factor is significant: any probabilities reported represent the posterior probability that a given variable had an odds ratio exceeding one.

For both approaches, we estimated a series of models including different sets of predictors. The first set (“patient” variables) included demographic characteristics. The second set (“pat. + comorbidities”) supplemented the background variables with patient comorbidities. The third set (“admission”) supplemented the previous with the initial measurements for the laboratory tests. The final regression (“post-admission”) then included percentage changes in each laboratory value from that at admission. See Section 3.3.2 for a description of these variable sets.

## 3. Results

### 3.1. Patient Characteristics

We collected data for 553 patients. Here, we describe the patient characteristics prior to data processing required for estimation.

The cohort consisted of 342 discharged patients and 211 expired patients. There were 271 (50.3%) females and 268 (49.7%) males. The median age was 69 years (range: 6–101 years). Most of the patients hospitalized were black (*n* = 472, 86.8%; Table 1).

The most common comorbidity was hypertension (*n* = 350, 64.0%) followed by diabetes (*n* = 229, 41.9%), hyperlipidemia (*n* = 103, 18.8%), end-stage renal disease (*n* = 54, 9.9%) and coronary artery disease (CAD) (*n* = 44, 8.0%). There was considerable within-patient clustering of the comorbidities (Figure S1).

We defined the test values at admission as the mean of those taken during the first day of hospitalization (Table S1). At admission, most patients had marked increase in CRP (median: 149 mg/L, Interquartile range (IQR): 80–246 mg/L), LDH (median: 468 IU/L, IQR: 342–638 IU/L) and ferritin (median: 879 ng/mL, IQR: 415–2132 ng/mL) levels. The patients also had decreased lymphocyte percentage (median: 12%, IQR: 8–16%). Patients also tended to have increased blood urea nitrogen (BUN; median: 27 mg/dL, IQR: 16–50 mg/dL).

### 3.2. Characteristics of Discharged and Expired Patients

**Figure 2:**
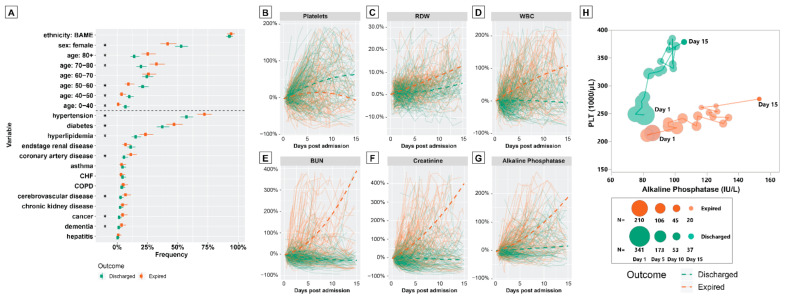
(**A**) Characteristics of patients according to outcome: the points indicate posterior median frequencies and the upper and lower whiskers indicate 2.5% and 97.5% posterior quantiles, calculated assuming a uniform prior and binomial likelihood. Asterisks indicate that the overlap in posteriors for each outcome was less than 0.05 for a given variable. (BAME refers to patients of non-Caucasian ethnicity.) (**B**–**H**) Time-series graphs of select laboratory values in hospitalized COVID-19 patients: Panels (**B**–**G**) show individual patient trajectories (solid lines) colored according to outcome for a selection of tests; dashed lines show fitted regression lines (see supplementary materials) for patients grouped by outcome). Panel (**H**) shows daily averages of platelets count (vertical axis) versus alkaline phosphatase levels (horizontal axis) for all patients remaining in hospital during the first 15 days of hospitalization. Each point indicates the paired mean of both tests for one of the two patient groups. (CHF: congestive heart failure, COPD: chronic obstructive pulmonary disease, N: number).

Expired patients were generally older (median: 73 versus 65 years old; $t_{540}=7.4,\;p<0.01$), more likely to be male (57% versus 45%: posterior overlap p<0.01) and to have hypertension, diabetes, hyperlipidemia, CAD, cerebrovascular disease, cancer or dementia (Figure 2A).

For 27 of 38 tests considered, there were significant differences in the initial test values between the discharged and the expired patients (Table S2). Notably, expired patients had higher CRP (median: 192 versus 124 mg/L) and LDH levels (median: 551 versus 427 IU/L), and lower lymphocyte percentage (median: 10.4% versus 12.8%), platelets (median: 188 versus 223 10^3/µL) and albumin levels (median: 3.3 versus 3.5 g/dL).

We then compared the trends in test values throughout patients’ hospitalization courses and analyzed the trends in test values as percentage changes from the values upon admission. To do so, we determined trends for patients grouped by outcome separately using linear mixed effects models (see SOM). Most tests showed visually distinct time trends for the two groups, indicating that, throughout hospitalization, those who went onto survive diverged from those who would not (Figure 2B–G, Figure S3). For example, patients who expired displayed less marked increases in platelet levels over time (Figure 2B) but exhibited relative increases in red cell distribution width (RDW; Figure 2C), white blood cell (WBC; Figure 2D), BUN (Figure 2E), creatinine (Figure 2F) and alkaline phosphatase (ALKP; Figure 2G) levels. Plotting the average tests scores for platelets and ALKP over time shows that it is possible to further separate survivors from those who died (Figure 2H). Indeed, this principle underpins the multivariate approach to determining risk factors in our dynamic Markov model.

### 3.3. Analysis of Dynamic Patient Risk

#### 3.3.1. Univariate Analysis

We used univariate Cox survival analyses [31] to illustrate the baseline patient characteristics and lab values upon admission that, individually, were the strongest determinants of risk within our sample (unadjusted). Increased age was the only statistically significant demographic risk factor (Table S3; Figure S2). From the comorbidities, only hyperlipidemia and CAD led to increases in mortality risk. Of the initial lab values, 17 were associated with mortality: the tests whose high values were most associated with mortality were total bilirubin (OR: 1.25), followed by mean platelet volume (OR: 1.15). Conversely, increased eosinophil percentage (OR: 0.67), large unstained cells percentage (OR: 0.81) and platelets level (OR: 0.99) were associated with lower mortality.

#### 3.3.2. Multivariate Analysis

**Figure 3:**
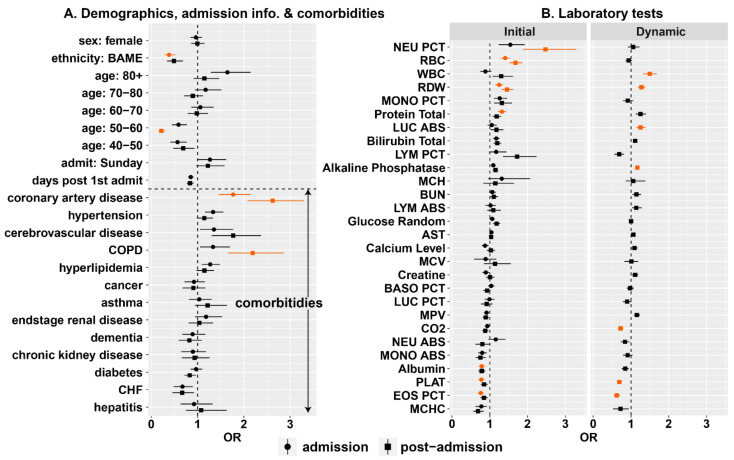
Estimated odds ratios for daily risk of death from Markov model: Panel (**A**) shows odds ratios (ORs) for demographic factors, variables associated with admission timing and comorbidities; Panel (**B**) shows ORs associated with lab values: both those upon admission (“Initial”) and % changes in lab values from these initial values (“Dynamic”). Marker types indicates the selection of regressors included in the model (see main text). Orange points indicate those odds ratios where the 5–95% posterior quantiles did not cross zero. Note, that the results for the lab values were obtained on data that had been standardized so that differences best reflect clinically relevant differences (see main text). (NEU PCT: neutrophils%, RBC: red blood cells, WBC: white blood cells, RDW: red cell distribution width, MONO PCT: monocytes%, LUC ABS: absolute large unstained cells count, LYM PCT: lymphocytes%, MCH: mean corpuscular hemoglobin, BUN: blood urea nitrogen, LYM ABS: absolute lymphocyte count, AST: aspartate aminotransferase, MCV: mean corpuscular volume, BASO PCT: basophils%, LUC PCT: large unstained cells%, MPV: mean platelets volume, NEU ABS: absolute neutrophil count, MONO ABS: absolute monocyte count, PLAT: platelets, EOS PCT: eosinophils%, MCHC: mean corpuscular hemoglobin concentration).

We used our Markov model with different combinations of variables to determine patient risk at admission and throughout the course of hospitalization. The multivariate results indicate fewer variables had a strong impact on risk of death; by controlling for more factors, certain variables, for example, age and sex, become less important predictors (Figure 3; Figure S4). Contrastingly, some factors, like CAD and chronic obstructive pulmonary disease (COPD), became more important. Similar results were also obtained by logistic regression analysis (Table S4), which considered only patients’ outcomes: not the time taken for the outcome to occur. Overall, the multivariate analyses show that there is substantial redundant information in the various factors, and only a relative few contain relevant independent information. 

Controlling for all the variables available upon admission, individuals aged over 80 were at higher risk (Figure 3A, circular markers; Table S5; OR: 1.64, Pr(OR>1)=0.92). Upon admission, CAD (OR: 1.77, Pr(OR>1)=0.98), cerebrovascular disease (OR: 1.35, Pr(OR>1)=0.80) and COPD (OR: 1.33, Pr(OR>1)=0.81) were the comorbidities most associated with elevated mortality risk.

To ensure that our odds ratios across different lab tests upon admission were on a comparable scale, we standardized data for each test meaning that odds ratios (shown in Figure 3B) are associated with the degree to which a value is above the mean and relative to the standard deviation (Table S6). Consistent with previous studies, upon admission, high values of certain tests were associated with a lower risk of death including albumin (>3.4 g/dL, OR: 0.79, Pr(OR>1)=0.04); platelets (>236,000/μL, OR: 0.78, Pr(OR>1)=0.03); eosinophil percentage (>0.64%, OR: 0.76, Pr(OR>1)=0.03) and mean corpuscular haemoglobin concentration (MCHC; >30.9%, OR: 0.78, Pr(OR>1)=0.15). For other variables, high values were associated with increased risk: neutrophil percentage (>79.4%, OR: 1.55, $Pr\left(OR>1\right)\;=0.92$); red blood cell count (RBC; >4.47 × 10^6^ cells/μL, OR: 1.41, $Pr\left(OR>1\right)\;=1.00$); monocyte percentage (MONO PCT; >5.0%, OR: 1.26, $Pr\left(OR>1\right)\;=0.89$); red cell distribution width (RDW; >14.9%, OR: 1.25, $Pr\left(OR>1\right)\;=0.96$) and total bilirubin (>0.74 mg/dL, OR: 1.17, $Pr\left(OR>1\right)\;=0.92$).

After a patient has been admitted, the predictive factors change as the results of ongoing laboratory tests are included (Figure 3, square markers). At this time, many of the variables most important at admission become less important predictors, for example, being older than 80 years is less predictive (OR: 1.15, $Pr\left(OR>1\right)\;=0.66$). Other variables, however, became more important predictors including CAD (OR: 2.62, Pr(OR>1)=1.00) and COPD (OR: 2.19, Pr(OR>1)=0.97). Many of the lab tests found important at admission continued to be so: high values of neutrophil percentage (OR: 2.48, Pr(OR>1)=0.99), RBC (OR: 1.68, Pr(OR>1)=1.00) and RDW (OR: 1.46, Pr(OR>1)=0.99) were associated with inflated mortality risk. Some of the admission values became more predictive, for example, high lymphocyte percentage was associated with higher risk (if >12.7%, OR: 1.72, Pr(OR>1)=0.94). Additionally, in the dynamic analysis, the risk of mortality increased the longer a patient was hospitalized (OR: 1.36, Pr(OR>1)=0.97).

In our analysis, we tracked the percentage change in each lab value relative to that at admission for each patient. Since different tests exhibited different dynamics over time (Figure S2), we standardized these percentage changes so that the odds ratios estimated across the different tests were on a comparable scale (Table S6). For a number of tests, above average increases in values over time raised the chance of death including: white blood cell count (OR: 1.5, Pr(OR>1)=0.99), RDW (OR: 1.28, Pr(OR>1)=0.99), total protein (OR: 1.25, Pr(OR>1)=0.92), the absolute count of large unstained cells (LUC ABS; OR: 1.25, Pr(OR>1)=0.95), bilirubin total (OR: 1.11, Pr(OR>1)=0.95) and ALKP (OR: 1.17, Pr(OR>1)=0.97). Above average changes in other variables signaled lower risk including: EOS PCT (OR: 0.62, Pr(OR>1)=0.00), platelets (OR: 0.69, Pr(OR>1)=0.01) and CO2 (OR: 0.73, Pr(OR>1)=0.01).

To assess the internal validity of the Markov model [23], we performed k-fold cross-validation (see SOM). We performed this analysis for each of the four regressions performed. The first three regressions offered similar levels of mean accuracy: the *patient* regression was able to determine the patient outcome with an accuracy of 67% (Figure S5); the *pat. + comorbidities* regression had an accuracy of 64%; and the *admission* regression had an accuracy of 67%. Including dynamic test values boosted predictive power to an accuracy of 83%. These results suggest that patient prognosis should be based on multiple factors rather than individual variables such as age.

### 3.4. Dynamic Measure of Risk

**Figure 4:**
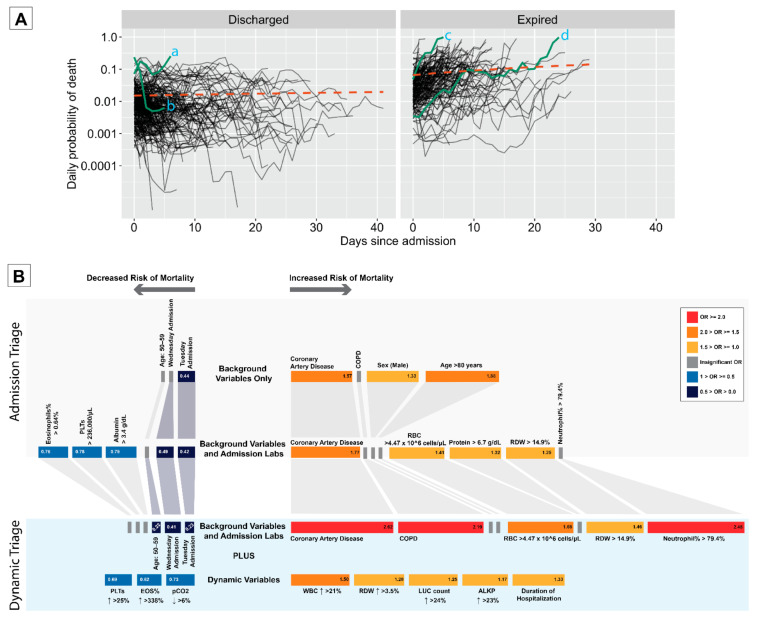
(**A**) Dynamic risk estimates for individual patients: Solid lines represent individual patients from time of admission until either their discharge (left) or death (right). Orange lines represent linear regression lines fitted on the linear scale. Vertical axis represents estimated daily probability of death during hospitalization (log-scale), which were obtained using the posterior median parameter values from the Markov model with *post-admission* regressors. Individual *a* was female aged over 80 with a history of hypertension and, generally, throughout the course of her stay was indicated to have a heightened risk of death: she was eventually discharged. Individual *b* was a male also aged over 80 with no recorded comorbidities: over time, his lab values changed substantially meaning their risk declined precipitously until his discharge. Individual *c* was female between 70 and 80 years old with a history of hypertension, hyperlipidemia, diabetes and coronary artery disease: over time, her values dramatically worsened, and she died five days after admission. Individual *d* was male aged between 50 and 60 years old and had end-stage renal disease, hypertension and diabetes: while his test values upon admission suggested low mortality risk, over time, his values worsened, and he died after more than 20 days in hospital. (**B**) Clinical decision tool for hospitalized COVID-19 patients: Each row of the diagram illustrates the key variables most useful for patient risk stratification based on the levels of available information. The block sizes, color and numbers within blocks represent odds ratios representing risk of daily mortality. The lab values indicate the cutoff point and direction of change associated with the given odds ratio.

We determined a dynamic measure of patient risk throughout hospitalization based on the Markov model which incorporated dynamic laboratory measurements. The model identified differences between the two groups upon admission: the group who went on to eventually be discharged had a mean probability of death on the first day of 0.015, whereas the expired group had a corresponding value more than four times greater. Throughout the course of hospitalization, the model could more accurately differentiate these groups, with the regression line for the discharged group remaining flat and that for the expired group increasing (Figure 4A)—highlighting the importance of accounting for dynamic test values. Despite this, it was not possible to perfectly predict patient outcomes due to the strong overlap in estimated probabilities between the groups (see selected individuals in Figure 4A).

### 3.5. Dynamic Triage

To facilitate decisions about risk stratification, we bring together the results of the Markov models into a clinical decision tool. This tool uses the most important factors for risk stratification dependent on the data available to clinicians at each time point. The decision tool is shown in Figure 4B.

## 4. Discussion

In this retrospective cohort study, we calculated the daily mortality risk of patients hospitalized with COVID-19 by developing a Bayesian Markov model that uses patient characteristics, including demographic variables, comorbidities, biomarkers at admission and time-dependent biomarkers. Our results suggest that solely relying on admission variables had limited accuracy and that only a handful of factors contributed to predictive accuracy (in line with current evidence [11]). Contrastingly, incorporating into our model dynamic variation in biomarkers measured throughout hospitalization led to dramatic improvements in predictive power. The sequential approach we propose provides clinicians with a tool that enables decision making depending on the level of information available [32].

Since the beginning of the COVID-19 outbreak, numerous decision support tools for patient prognosis have been developed [11,12]. Given the reactive nature of the pandemic response, prognostic modelling efforts have suffered from a number of limitations, including lack of external validation and a high risk of bias [12]. We fit our model to data from a single hospital in New York, where our study population mostly consisted of black African American patients who have been disproportionately affected by the COVID-19 pandemic [33,34]. Socioeconomic variables may impact in-hospital mortality risk [21,35], and external validation of ours and other models is therefore required. Finally, any clinical implementation requires an assessment of the impact of the prognostic model on clinicians’ behavior, patient health and associated costs [10].

Our model allows for risk stratification and triage. At the patient level, the model allows for individualization of care for each hospitalized COVID-19 patient and identification of need for additional levels of care [36]; the main advantage of our approach is the incorporation of dynamic variables which allows for daily adjustments to the patient’s in-hospital mortality risk. In addition, by identifying high-risk patients and determining the surge capacity needed for advanced intensive care, our approach could allow for early resource allocation and ultimately improved outcomes for patients [17,37]. However, with insufficient surge capacity, triage should consider both patient prognosis and ethical considerations to avoid health inequities [6,38].

Currently, COVID-19 hospitalization rates remain high in many countries, and it is imperative that patients receive the best care available, and prognostic models like ours likely have a role to play in achieving this. Whilst our approach was applied to severely ill COVID-19 patients, similar patterns of inflammatory response and multi-organ injury are also seen in other acutely ill patients [39,40]. Identifying the effect of dynamic changes in relevant biomarkers on mortality risk in real time, using a tool like the one we develop, could be transformative in caring for these patients.

## Figures and Tables

**Table 1:** Summary of patient characteristics. Note, that a number of variables have missing observations meaning that the totals across all groups do not sum to the total number of patients (*n* = 553): for example, there were 14 patients whose sex was not recorded.

Category	Variable	Count	Frequency
Outcome	Outcome: Discharged	342	61.8%
	Outcome: Expired	211	38.2%
Demographic	Sex: Female	271	50.3%
	Sex: Male	268	49.7%
	Age: 0–40	26	4.8%
	Age: 40–50	43	7.9%
	Age: 50–60	93	17.2%
	Age: 60–70	140	25.8%
	Age: 70–80	137	25.3%
	Age: 80–100	103	19.0%
	Ethnicity: Black	472	86.8%
	Ethnicity: Caucasian	25	4.6%
	Ethnicity: Hispanic	17	3.1%
	Ethnicity: Other or Unrecorded	39	7.1%
Comorbidities	Asthma	24	4.4%
	Cancer	16	2.9%
	Cerebrovascular Disease	25	4.6%
	Congestive Heart Failure	23	4.2%
	Chronic Kidney Disease	19	3.5%
	Chronic Obstructive Pulmonary Disease	25	4.6%
	Coronary Artery Disease	44	8.0%
	Dementia	13	2.4%
	Diabetes	229	41.9%
	End-Stage Renal Disease	54	9.9%
	Hepatitis	4	0.7%
	Hyperlipidemia	103	18.8%
	Hypertension	350	64.0%

## Supplementary Materials

The following are available online at https://www.mdpi.com/2036-7449/13/1/27/s1, Table S1: Summary of test values upon admission, Figure S1: Comorbidity correlations, Table S2: Comparison of admission test values between discharged and expired patients, Table S3: Univariate Survival Analysis of baseline characteristics and admission test values, Figure S2: Kaplan-Meier survival curves for select variables, Table S4: Logistic model results, Table S5: Markov model results, Figure S3: Trends in lab values, Figure S4: Markov model: estimated odds ratios indicating mortality risk, Figure S5: Markov model: predictive accuracy, Figure S6: Posterior predictive check: simulated versus actual mortality by age group, Figure S7: Posterior predictive check: simulated (black) and actual (orange) mortality across different subgroups, Figure S8: Posterior predictive check: simulated (black) and actual (orange) mortality according to last observed change in lab values, Figure S9: Posterior predictive check: simulated versus actual hospitalization duration, Table S6: Summary statistics of lab values, Table S7: Priors for Markov model parameters, [41,42,43,44,45,46].
